# Real clinical experience of inhaled esketamine in psychotic depression. A retrospective study

**DOI:** 10.1192/j.eurpsy.2025.1304

**Published:** 2025-08-26

**Authors:** S. Benavente López, E. Toro Carrasco, I. Pedrero Torrejon, A. Lara Fernández, A. Parra González, M. Mejia Quiterio, A. Viñas Arboleda, E. Baca García

**Affiliations:** 1 Department of Psychiatry, Hospital Universitario Infanta Elena, Madrid, Spain

## Abstract

**Introduction:**

Psychotic depression is a severe mental disorder that can cause severe suffering and dysfunctionality in the patient. It is characterised by the presence of major affective symptoms together with the presence of psychotic symptoms in the form of delusions and/or sensory-perceptual alterations. Psychotic depression is particularly severe when it becomes resistant to pharmacological treatment, often requiring the combination of antipsychotics with antidepressants and even the use of electroconvulsive therapy. Lifetime prevalence of psychotic depression ranges from 0.35% to 1%, being more frequent in the elderly. Previous studies have shown positive experience with intravenous ketamine in the treatment of psychotic depression, improving psychotic symptomatology and showing a good safety and tolerability profile. The present study shows the response in a series of cases with psychotic depression treated with inhaled esketamine, showing their evolution through the Montgomery-Asberg Depression Rating Scale (MADRS) as well as their safety and tolerability profile.

**Objectives:**

The main objective of this study is to describe the use of inhaled esketamine in patients with psychotic depression in real clinical practice, using the MADRS as main outcomes to show the evolution.

**Methods:**

Retrospective descriptive study with a sample selected by non-probabilistic consecutive sampling, retrospective type, in a time interval of 2 years. The patients selected were those who completed treatment with inhaled esketamine and had a diagnosis of psychotic depression at Hospital Universitario Infanta Elena. A descriptive analysis was performed. Mean and standard deviation were calculated for quantitative variables and N and percentage for categorical variables.

**Results:**

A total of 5 patients with a diagnosis of psychotic depression (n: 5) all treated with inhaled esketamine were included in the study. The efficacy of inhaled esketamine was very high in the treatment of patients with psychotic depression, achieving a 100% response rate (MADRS<10). All 5 patients showed a rapid improvement in psychotic symptomatology, with cessation of psychotic symptoms in the initial phases of treatment, showing a rapid response. No worsening of psychotic symptoms was observed at any time with the administration of inhaled esketamine. Tolerability was excellent, with no serious adverse effects occurring during treatment administration.

**Conclusions:**

The present study shows a high efficacy of esketamine in the treatment of psychotic depression, with a high response rate and an excellent tolerability profile. Inhaled esketamine could be a useful tool in the treatment of psychotic depression, helping to rapidly restore patients’ functionality. Longitudinal studies must be carried out to confirm this hypothesis.

**Disclosure of Interest:**

None Declared

